# Similar 2 year migration patterns for a shortened and a distally reduced stem design in uncemented total hip arthroplasty: a randomized controlled trial using radiostereometric analysis

**DOI:** 10.2340/17453674.2026.46551

**Published:** 2026-08-31

**Authors:** Lennard A KOSTER, Bart L KAPTEIN, Esmeralda GOORDEN, Constant A M P BELL, Rob G H H NELISSEN

**Affiliations:** 1 Department of Orthopaedics, Leiden University Medical Center, Leiden; 2 Department of Orthopaedic Surgery, Bravis Ziekenhuis, Roosendaal, the Netherlands.

## Abstract

**Background and purpose:**

The primary objective was to compare subsidence and retroversion in the first 2 postoperative years of 2 uncemented hip stem designs in primary minimally invasive anterior-supine surgical (MIS-ASI) total hip arthroplasty: a shortened hip stem (Microplasty) and a standard-length stem with reduced distal width (Reduced Distal). Secondary objectives were to compare migration patterns, patient-reported outcome measures (PROMs), clinical and radiological scores, and the occurrence of (serious) adverse events up to 2 years postoperatively.

**Methods:**

50 patients were randomized in this non-blinded, single-surgeon, industry-funded, radiostereometric analysis study (RSA) (ClinicalTrials.gov NCT03409666). Stem migration was measured relative to directly postoperative at 6 weeks, 1 year, and 2 years postoperatively, using model-based RSA. Linear mixed-effect modelling was used to compare migration. Primary outcomes were subsidence and retroversion at 2 years postoperatively. Secondary outcomes were migration, PROM questionnaires, clinical and radiological scores, and the presence of adverse events in the first 2 postoperative years.

**Results:**

Migration of 15 Microplasty and 20 Reduced Distal stems showed comparable initial migration between direct and 6 weeks postoperative RSA, stabilizing thereafter. Predominant migration directions were subsidence and retroversion with mean values at 2 years of 1.47 mm (95% confidence interval [CI] 0.32–2.61) and 2.57° (CI 1.64–3.50) for the Microplasty stem and 2.35 mm (CI 1.36–3.34) and 1.59° (CI 0.79–2.39) for the Reduced Distal stem. At 6 weeks postoperatively, posterior tilt about the medial–lateral axis was different between stem designs, with 0.07° (CI –0.27 to 0.40) for the Microplasty and 0.48° (CI 0.19–0.77) for the Reduced Distal designs. Secondary outcomes were not clinically different between the stem designs.

**Conclusion:**

We showed that initial subsidence and retroversion for both stem designs in MIS-ASI surgery was present, although likely not clinically relevant. There was no statistically significant difference in subsidence and retroversion between the stem designs. Although initial migration varied substantially between patients and the Reduced Distal design showed more posterior tilting, both designs maintained a stable position from 6 weeks to 2 years postoperatively.

Primary total hip arthroplasty (THA) is a proven procedure to restore hip function and to reduce pain in patients with end-stage hip disabling disease. Minimally invasive surgery (MIS) in combination with an anterior-supine-intermuscular (ASI) approach in THA has gained in popularity as it may reduce soft tissue damage and shorten the rehabilitation period [1]. However, MIS-ASI provides less visibility and working space around the hip joint and therefore alternative stem designs suitable for MIS-ASI THA have been developed.

2 variations of an existing hip stem were developed for use in MIS, a shortened stem (Microplasty) and a stem with the same length, but a reduced distal width (Reduced Distal). Shorter stems were found to be associated with poorer outcomes [2], though a recent large registry study showed comparable group 10-year overall survival for short and standard length stems, with aseptic loosening as the main reason for short stem revision [3]. Evidence on implant fixation and clinical outcomes of the Microplasty and Reduced Distal stems from non-designer research groups is scarce [4-7]. Radiostereometric analysis (RSA) is a proven technique that can be used to predict the long-term risk for aseptic loosening of orthopedic implants [8]. For uncemented THA the consensus is that stabilization of the stem is an important factor for a small risk of aseptic loosening [9,10].

At present, there is no study comparing migration of the Microplasty and the Reduced Distal hip stem designs using the MIS-ASI technique. Therefore, the primary objective was to compare stability of 2 uncemented hip stem designs in THA using the MIS-ASI technique in the first 2 postoperative years using RSA measured migration patterns. Primary outcome variables were subsidence and retroversion at 2 years postoperatively, hypothesizing no difference between the 2 designs. Secondary objectives were to compare migration patterns, patient-reported outcome measures (PROMs), clinical and radiological scores, and the occurrence of (serious) adverse events up to 2 years postoperatively.

## Methods

### Study design

A non-blinded randomized controlled trial including patients with either the shortened (Taperloc Complete Microplasty Reduced Distal [Microplasty]) or standard length with reduced distal width (Taperloc Complete Reduced Distal [Reduced Distal]) stem (both Zimmer Biomet, Warsaw, IN, USA) was performed in a general hospital (Bravis Ziekenhuis, Breda, the Netherlands).

The study protocol was written by the sponsor (Zimmer Biomet). The sponsor provided funds to the authors’ institutions for the RSA study. Study management was performed by the hospital and sponsor. Study data was collected by the hospital and entered into the sponsor’s study management system. All study data was provided to the authors for analysis. Patient and public involvement was not part of the design, conduct, and reporting of the study.

In ClinicalTrials, maximum total point motion (MTPM) was registered as the main outcome variable by the study sponsor. However, subsidence and retroversion of hip stems are generally considered to be the most relevant migration variables [9,10]. Therefore, we chose to use these variables as the main outcome variables in the study. This decision, although not registered in a protocol amendment, was made by the authors before having analyzed the results.

Though this study did not meet all the criteria, reporting of the trial is as much as possible in line with the CONSORT statement and ICMJE guideline.

### Patients and surgery

Patients eligible for primary THA were invited by the treating physician to participate in the study. Inclusion criteria were patients with end-stage non-inflammatory degenerative joint disease, between 18 and 70 years of age. Patients with any sign of active infection, metabolic disorders, neuromuscular disease, vascular insufficiency, or osteonecrosis were excluded.

Surgeries, all for end-stage osteoarthritis, were performed between June 2017 and September 2018 using the MIS-ASI technique by the same surgeon (CB), who had longstanding experience with this surgical technique using both stem designs.

**Figure 1:**
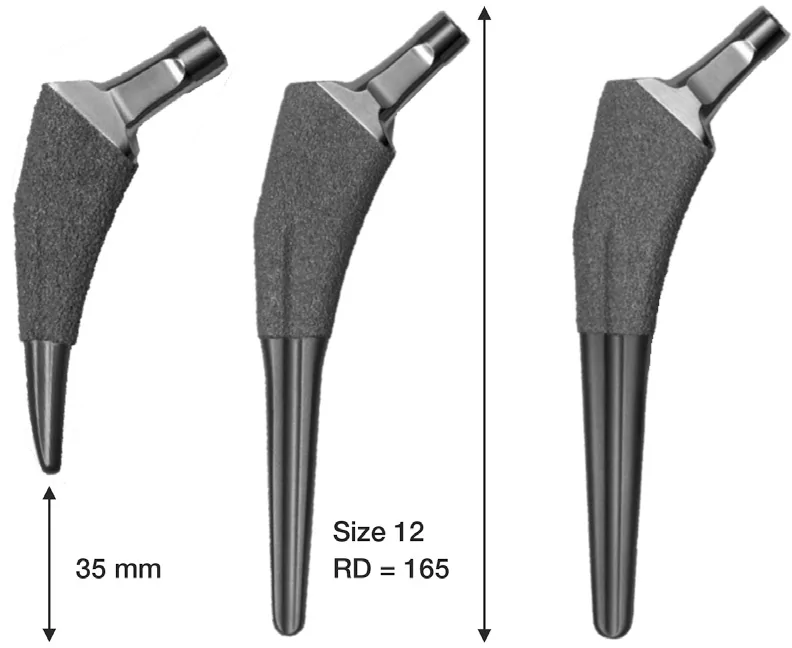
Study stems are the Taperloc Complete Microplasty (Microplasty, left) and Taperloc Complete Reduced Distal (Reduced Distal, middle) stems. For comparison, the Taperloc Complete Full Profile (right) stem (not included in the study) is shown. Figures not scaled and with different view angles. All stems in the study have a 133° caput–collum–diaphyseal (CCD) angle.

Both titanium stems are variations of the standard uncemented Taperloc Complete full profile design, with an identical proximal geometry, including the titanium porous plasma spray coating with hydroxyapatite coating. The stem diameter distally of the coated part of both designs is smaller than the standard stem, and for the Microplasty stem this part is 35 mm shorter than the standard and Reduced Distal stems (Figure 1). The caput–collum–diaphyseal (CCD) angle of the stems was 133°. Both stems were equipped with a Biolox Delta ceramic femoral head in combination with an uncemented acetabular cup (G7 OsseoTi cup with a vitamin-E polyethylene liner (all Zimmer Biomet, Warsaw, IN, USA).

Rehabilitation was according to the hospital’s standard rapid recovery program with full weightbearing as tolerated starting on the day of surgery or the first postoperative day.

### Primary outcome

Primary outcomes of this study were RSA measured subsidence and retroversion at 2 years postoperatively. Other RSA measured migration variables were secondary outcomes, but for clarity of the methods sections these are described in this section as well.

For RSA, at least 5 tantalum markers (1 mm diameter) were inserted intraoperatively into the femoral bone before insertion of the stem. After weightbearing on the operated hip, supine RSA radiographs were scheduled to be made on the first postoperative day (baseline), then postoperatively at 6 weeks, 1 year, and 2 years postoperatively. At 1 year a double RSA acquisition was made to determine the clinical precision (Table 1). 2 roentgen tubes (DigitalDiagnost and MobileDiagnost CR Systems, Philips Medical Systems, the Netherlands) were used in combination with a uniplanar calibration cage (CageID HBI012, Halifax Biomedical Inc, Mabou, Halifax, Canada) and 2 35×43-cm detectors. RSA radiographs, 127 dots per inch, were sent to RSA*core* (LUMC, the Netherlands) for analysis. Model-based RSA software (version 4.2, RSA*core*, LUMC, the Netherlands) using computer-aided design (CAD) models [11,12] was used for model-based RSA analysis. Translations and rotations were determined at each timepoint with respect to the geometric center of the stem model at baseline. The point at the component that moved most between baseline and follow-up was registered as MTPM. For each patient, the largest consistent set of markers [13] available in all RSA radiographs was used, with a minimum of 3 markers, meeting the criteria for rigid-body matching (mean error [ME] ≤ 0.35 mm; condition number [CN] ≤ 150) [14]. For 5 hips (1 Microplasty, 4 Reduced Distal) a marker-configuration (MC) model was created to meet the CN threshold [15]. Information concerning the RSA measurements (e.g., numbers of markers used, CN, mean error of rigid-body fitting) is available in Table S4 (see Supplementary data).

**Table 1:** Clinical precision of RSA measurements based on double examinations acquired 1 year postoperatively

Implant	Translation (mm)			Rotation (°)		
	X	Y	Z	X	Y	Z
Microplasty (n = 15)						
Mean	–0.01	0.04	0.01	0.06	0.13	–0.02
SD	0.05	0.13	0.17	0.25	0.51	0.12
Reduced Distal (n = 20)						
Mean	0.01	0.01	0.02	0.01	0.04	–0.03
SD	0.10	0.11	0.21	0.35	0.57	0.09

X, Y, Z = orthogonal axes (X = transverse axis, Y = longitudinal axis, Z = sagittal axis); SD = standard deviation.

**Figure 2:**
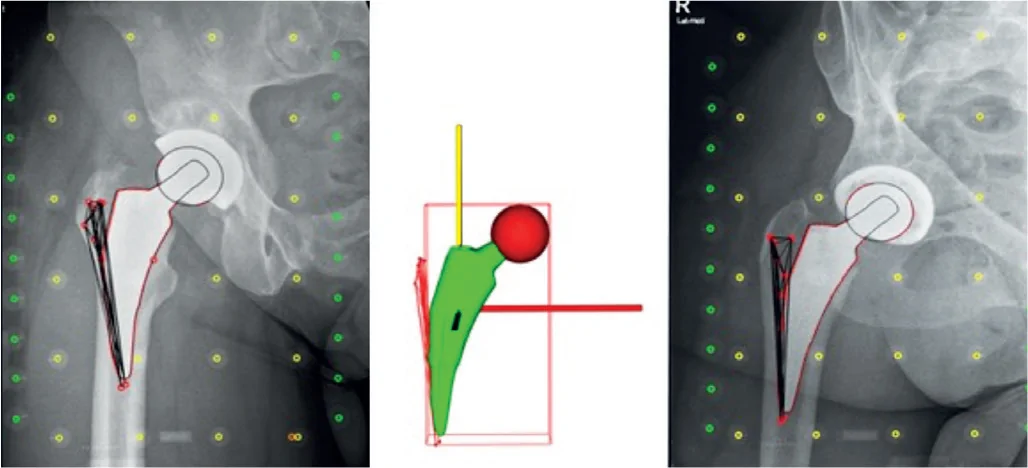
RSA scene and the orientation of the migration coordinate system: red line is the X-axis, yellow is the Y-axis, and the Z-axis is out of plane pointing towards the viewer.

For migration analysis, results from left-sided hip stems were converted to a right-sided hip [8]. In this study results are presented for a right-sided stem with positive translation in millimeter (mm) medially along the transverse axis (Tx), proximally along the longitudinal axis (Ty; subsidence is negative Ty), and anteriorly along the sagittal axis (Tz). Positive rotations in degrees (°), according to the right hand rule, are anterior tilt (Rx), retroversion (Ry), and varus rotation (Rz) [8] (Figure 2).

### Secondary outcomes

Secondary outcomes were collected prior to surgery, immediately postoperatively, and at 6 weeks, 1, and 2 years postoperatively. The following PROMs were collected: Hip disability Osteoarthritis Outcome Score (HOOS), EuroQol 5-dimension (EQ-5D-3L), Oxford Hip Score (OHS), and Forgotten Joint Score (FJS, only postoperative). At each postoperative visit thigh pain and Harris Hip Score (HHS) were scored by the treating surgeon. Adverse events and serious adverse events were registered at any time during follow-up.

Routine anteroposterior and lateral hip radiographs were used to score canal flare index (CFI) [16], implant sizing, and alignment (stem axis related to diaphyseal axis: < 5° deviation = varus/valgus; > 5° deviation = severe varus/valgus; no deviation = neutral), metaphyseal filling [17], presence of fractures, periprosthetic radiolucent lines (RLL), and signs of stress shielding [18,19]. RLL > 3 mm were considered as radiographically failed implant fixation.

### Statistics

For this study it was decided to include 25 patients in each group to compare stem stability and migration over time, assuming equivalence. This number of required patients is stated in the RSA standardization paper from 2005 to be sufficient for RSA studies [14]. Randomization (random number generator, using equal block sizes of 10) in a 1:1 ratio was performed by an independent study nurse, retrieving the next randomly generated group assignment prior to templating of required implant size.

Primary and secondary outcome variables were evaluated using SPSS (v25, IBM Corp, Armonk, NY, USA) and R v. 1.2.1335 (R Foundation for Statistical Computing, Vienna, Austria, with packages nlme, emmeans, dplyr, plotly, ggplot2). Assuming linearity and homogeneity of variance, linear mixed-effects modelling (LMM) [20] was applied to compare migration over 2 years and to compare migration rate between 6 weeks and 2 years postoperatively. The lme() function from the nlme package in R was used with stem design, time, and their interaction set as fixed effects, and each hip was included as random effect. CorCAR(1), continuous autoregression-1, was selected to model within-subject correlation over time. Residuals of the LMM were visually inspected for normality (Q–Q plots and Shapiro–Wilk test). MTPM results were log-transformed before LMM and back-transformed for presentation of migration values. Migration results from the LLM, including MTPM, although not normally distributed, are presented as estimated means with 95% confidence intervals (CI) and estimated mean difference between stem designs. Observed PROM results are presented as medians with interquartile ranges (IQR). As subsidence of the stem was the main outcome, analysis was performed by implanted stem design (as treated with Microplasty or Reduced Distal; hence no intention-to-treat analysis), using all available data. Group results are based on LMM modelling. Unmodelled migration data is shown in Tables S1A and S1B (see Supplementary data).

### Ethics, data sharing plan, funding, use of AI, and disclosures

The study was performed in compliance with the Declaration of Helsinki (2018) and Good Clinical Practice guidelines (ICH-GCP R2). The medical ethics committee METC Leiden-Den Haag-Delft approved the study (23 December 2016, ABR NL54031.098.15), and the study was registered in Clinical Trials (January 8, 2018, ClinicalTrials.gov ID NCT03409666). All patients gave written informed consent prior to inclusion.

The study was conceptualized by Zimmer Biomet. Zimmer Biomet provided funds to the authors’ institutions related to the costs associated with this RSA study. None of the authors, nor any member of their immediate families, received funding or have commercial associations (e.g., consultancies, stock ownership, equity interest, patent/licensing arrangements, etc.) that might pose a conflict of interest in connection with the submitted manuscript. Zimmer Biomet helped with the study design and data collection, but did not take part in the analysis, interpretation, or writing of the manuscript. Complete disclosure of interest forms according to ICMJE are available on the article page, doi: 10.2340/17453674.2026.46551

## Results

**Figure 3:**
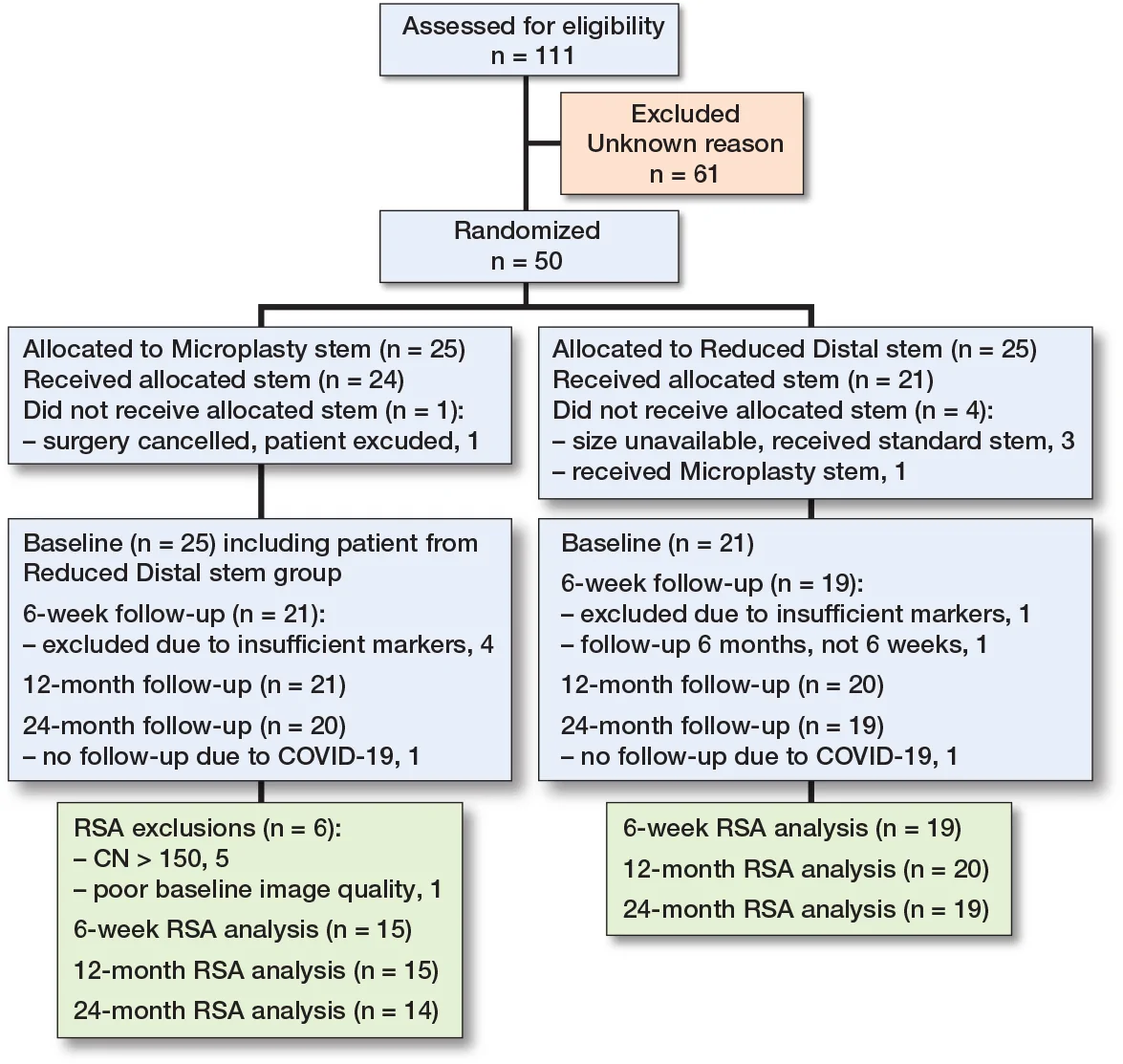
Consort flow diagram. CN = condition number. The reasons for exclusion were not meeting inclusion criteria, having met an exclusion criterium or not giving consent. However the numbers for each reason were lost in a departmental move.

Patient enrollment is provided in the flowchart (Figure 3). Of 111 eligible patients screened, 61 did not meet the inclusion criteria, had an exclusion criterion, or did not provide informed consent. Thus, 50 patients were randomized. 4 patients were excluded from analysis after randomization: 1 patient withdrew from the study before surgery for personal reasons and for 3 patients the necessary stem size did not exist in the allocated design (< size 9).

During surgery 1 patient randomized to Reduced Distal received a Microplasty stem by mistake. All stems were in situ after 2 years.

Due to not meeting the RSA technical requirements as specified in the methods section, another 11 stems (10 Microplasty, 1 Reduced Distal) were excluded from RSA analysis, resulting in RSA data from 15 Microplasty and 20 Reduced Distal stems being available for analysis.

Preoperative patient characteristics and patient characteristics of the 2 groups were comparable (Table 2).

**Table 2:** Patient characteristics, as randomized and as implanted. Values are count or as specified

Factor	Randomized		Implanted **^a^**	
	Microplasty (n = 25)	Reduced Distal (n = 25)	Microplasty (n = 25)	Reduced Distal (n = 21)
Female sex	19	18	20	15
Age at surgery, mean (SD)	64 (6.5)	63 (7.0)	64 (6.3)	64 (6.5)
Body mass index, mean (SD)	28 (5.7)	27 (3.2)	28 (5.9)	27 (2.9)
Operated on left side	13	9	12	7
ASA score				
ASA 1	5	5	5	4
ASA 2	18	20	18	17
ASA 3	2	–	2	–
Canal Flare Index (CFI)				
Stovepipe (CFI < 3)	1	3	1	3
Normal (3 < CFI < 4.7)	23	20	23	17
Champagne-fluted (CFI > 4.7)	1	2	1	1
Operation time, minutes, mean (SD)	n.a.	n.a.	48 (8.5)	49 (9.3)

a 1 patient randomized to Microplasty withdrew before surgery; 3 patients randomized to Reduced Distal received a non-study stem, and 1 patient randomized to Reduced Distal received a Microplasty stem by mistake.

ASA = American Society of Anesthesiologists; n.a. = not applicable (as operation time is only provided after the actual surgery); SD = standard deviation.

### Primary outcome

**Figure 4:**
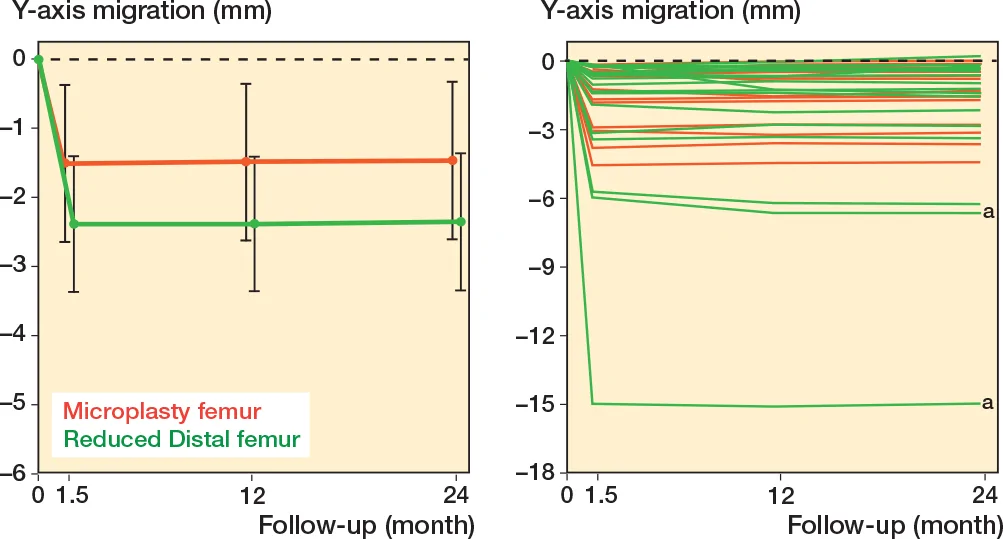
Y-axis translation (linear mixed model results) of Microplasty (red) and Reduced Distal (green) stems presented as mean group results with 95% confidence interval (left) and for individual stems (right) (as implanted). a = underfilled femoral canal.

**Figure 5:**
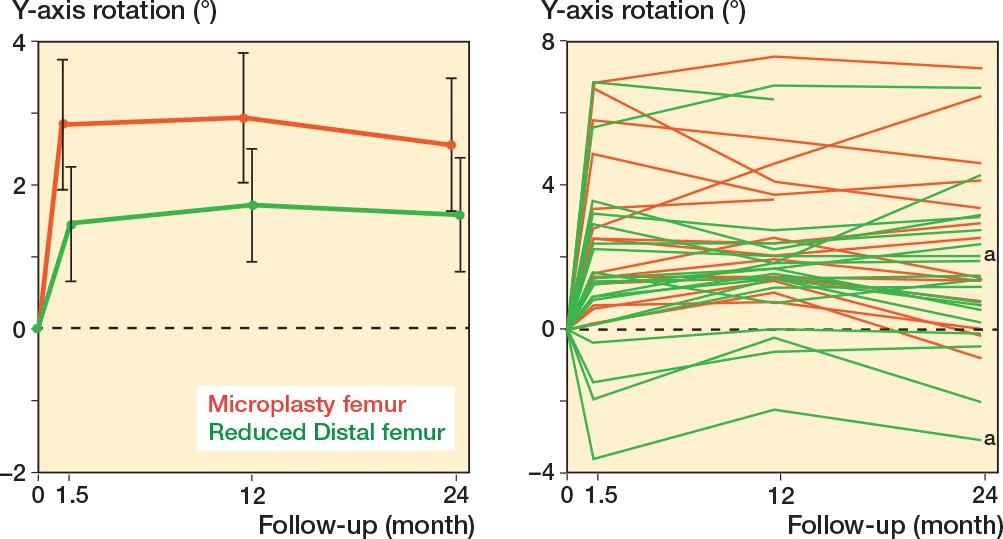
Y-axis rotation (linear mixed model results) of Microplasty (red) and Reduced Distal (green) stems presented as mean group results with 95% confidence interval (left) and for individual stems (right) (as implanted). a = underfilled femoral canal.

Migration occurred mainly as subsidence (negative Y-axis translation) and retroversion (positive Y-axis rotation) within the first 6 weeks postoperatively (Figures 4 and 5, Table 3). Mean Y-axis translation of the Microplasty design at 6 weeks was –1.51 mm (CI –2.64 to –0.38) and –2.38 mm (CI –3.37 to –1.40) for the Reduced Distal with a mean design difference of 0.88 mm (CI –0.73 to 2.48). Mean Y-axis rotation of the Microplasty design at 6 weeks was 2.85° (CI 1.94–3.76) and 1.46° (CI 0.66–2.25) for the Reduced Distal with a mean design difference of 1.39° (CI 0.10–2.68).

**Table 3:** Modelled (LMM) mean migration results with 95% confidence interval (CI) and estimated mean difference (CI) of 15 Microplasty and 20 Reduced Distal stems (as implanted) at 6 weeks’, 1 year, and 2 years’ follow-up, with respect to directly postoperative follow-up

Migration axis Follow-up	Mean migration (CI)		Mean difference (CI) (Microplasty – Reduced Distal)
	Microplasty group	Reduced Distal group	
Tx (mm)			
6 weeks	0.28 (–0.23 to 0.79)	–0.24 (–0.68 to 0.21)	0.52 (–0.20 to 1.24)
1 year	0.32 (–0.19 to 0.83)	–0.20 (–0.64 to 0.24)	0.52 (–0.20 to 1.24)
2 years	0.24 (–0.28 to 0.75)	–0.22 (–0.67 to 0.22)	0.46 (–0.27 to 1.19)
Ty (mm)			
6 weeks	–1.51 (–2.64 to –0.38)	–2.38 (–3.37 to –1.40)	0.88 (–0.73 to 2.48)
1 year	–1.49 (–2.62 to –0.35)	–2.38 (–3.36 to –1.40)	0.89 (–0.71 to 2.50)
2 years	–1.47 (–2.61 to –0.32)	–2.35 (–3.34 to –1.36)	0.88 (–0.74 to 2.51)
Tz (mm)			
6 weeks	–0.47 (–0.67 to –0.27)	–0.22 (–0.40 to –0.05)	–0.25 (–0.53 to 0.04)
1 year	–0.30 (–0.50 to –0.10)	–0.29 (–0.46 to –0.11)	–0.01 (–0.29 to 0.27)
2 years	–0.15 (–0.35 to 0.05)	–0.29 (–0.46 to –0.11)	0.13 (–0.15 to 0.42)
Rx (°)			
6 weeks	–0.07 (–0.40 to 0.27)	–0.48 (–0.77 to –0.19)	0.42 (–0.06 to 0.89)
1 year	0.00 (–0.33 to 0.33)	–0.60 (–0.88 to –0.31)	0.59 (0.12 to 1.06)
2 years	–0.06 (–0.28 to 0.39)	–0.56 (–0.86 to –0.27)	0.62 (0.14 to 1.10)
Ry (°)			
6 weeks	2.85 (1.94 to 3.76)	1.46 (0.66 to 2.25)	1.39 (0.10 to 2.68)
1 year	2.94 (2.03 to 3.85)	1.72 (0.94 to 2.51)	1.22 (–0.07 to 2.50)
2 years	2.57 (1.64 to 3.50)	1.59 (0.79 to 2.39)	0.98 (–0.33 to 2.29)
Rz (°)			
6 weeks	–0.34 (–0.76 to 0.08)	0.14 (–0.23 to 0.51)	–0.48 (–1.09 to 0.12)
1 year	–0.27 (–0.69 to 0.16)	0.12 (–0.25 to 0.49)	–0.39 (–0.99 to 0.21)
2 years	–0.31 (–0.74 to 0.12)	0.13 (–0.24 to 0.50)	–0.43 (–1.04 to 0.17)
MTPM **^a^** (mm)			Ratio (CI) **^b^**
6 weeks	3.05 (2.12 to 4.25)	2.79 (2.01 to 3.76)	1.07 (0.76 to 1.51)
1 year	2.97 (2.06 to 4.15)	3.12 (2.29 to 4.17)	0.96 (0.68 to 1.36)
2 years	2.68 (1.82 to 3.79)	3.04 (2.22 to 4.08)	0.91 (0.64 to 1.29)

LMM = linear mixed model; MTPM = maximum total point motion (mm); Tx, Ty, Tz = translation in millimeters (mm) along the X-, Y-, and Z-axes; Rx, Ry, Rz = rotation in degrees (°) about the X-, Y-, and Z-axes.

a Estimated mean MTPM for each group at each time point (back-transformed values from LMM).

b Ratio of the LMM difference on the log-transformed scale between the groups. At 6 weeks the value of 1.07 indicates that MTPM of the Microplasty group is 7% larger than that of the Reduced Distal group.

2 years after surgery mean (CI) subsidence and retroversion were 1.47 mm (CI 0.32–2.61) and 2.57° (CI 1.64–3.50) for the Microplasty stem and 2.35 mm (CI 1.36–3.34) and 1.59° (CI 0.79–2.39) for the Reduced Distal stem. The migration rate between 6 weeks and 2 years postoperatively showed stabilization for both stems in subsidence and retroversion (Table 4). The mean design difference for the migration rate of subsidence and retroversion was small and seemed to be similar: mean difference in subsidence was –0.04 mm (CI –0.11 to 0.03) and 0.27° (CI –0.09 to 0.63) for retroversion (Table 4).

**Table 4:** Mean migration rate (LMM results expressed per year) of the Microplasty and Reduced Distal stems with 95% confidence interval (CI) based on all available 6 weeks’ to 2 years’ postoperative RSA data of 15 Microplasty and 20 Reduced Distal stems (as implanted)

Migration axes	Mean annual migration (CI)		Mean difference (CI) (Microplasty – Reduced Distal)
	Microplasty group	Reduced Distal group	
Tx (mm/year)	–0.02 (–0.07 to 0.03)	0.02 (–0.03 to 0.06)	–0.04 (–0.10 to 0.03)
Ty (mm/year)	0.04 (–0.02 to 0.09)	0.00 (–0.05 to 0.05)	0.04 (–0.03 to 0.11)
Tz (mm/year)	0.17 (0.08 to 0.25)	–0.03 (–0.11 to 0.04)	0.20 (–0.06 to 0.32)
Rx (°/year)	0.05 (–0.06 to 0.17)	–0.07 (–0.17 to 0.03)	0.12 (–0.03 to 0.27)
Ry (°/year)	–0.15 (–0.42 to 0.12)	0.12 (–0.11 to 0.36)	–0.27 (–0.63 to 0.09)
Rz (°/year)	0.01 (–0.05 to 0.08)	–0.01 (–0.07 to 0.05)	0.02 (–0.06 to 0.11)

Tx, Ty, Tz = translation in millimeters (mm) along the X-, Y-, and Z-axes; Rx, Ry, Rz = rotation in degrees (°) about the X-, Y-, and Z-axes.

### Secondary outcomes

A difference between the designs appears to be present for rotation about the X-axis (Tables 3 and 4). At 6 weeks, the educed Distal design had rotated posteriorly, whereas the Microplasty did not rotate. This difference persisted at later follow-ups, with both stem designs stabilizing after initial migration (Table 4).

At 6 weeks’ follow-up, small initial differences between the groups were present in medial–lateral (X-axis) and anterior–posterior (Z-axis) translations as well as in internal–external (Y-axis) and varus–valgus (Z-axis) rotations (Table 3). The migration rate in Z-axis translation appeared to be different between the designs: the MP stem seemed to translate anteriorly (mean: 0.17 mm/year, CI 0.08–0.25), whereas the RD did not show anterior translation (mean: –0.03 mm/year, CI –0.11 to 0.04) and the mean difference between the groups was –0.20 mm/year (CI –0.32 to 0.06) (Table 4).

Clinical and patient-reported outcome measures all improved to near-optimal scores at 2 years’ follow-up for both groups (Supplementary Table S2).

Direct postoperative radiographic evaluation of the 25 Microplasty and 21 Reduced Distal implanted stems identified 9 varus aligned stems (< 5° varus, 4 Microplasty and 5 Reduced Distal), 4 valgus aligned stems (< 5° valgus, 2 Microplasty and 2 Reduced Distal), and 33 stems in neutral alignment (19 Microplasty and 14 Reduced Distal).

There were no cases of incorrect stem sizing registered in relation to the femoral bone anatomy, 4 registrations of thigh pain (all at 6 weeks; 1 Microplasty and 3 Reduced Distal), no intra- or postoperative fractures, and no RLL were detected for any of the patients. Subsidence was observed on plain radiographs for 8 stems (2 Microplasty and 6 Reduced Distal). Median (range) RSA subsidence for these patients was 5.5 mm (2.1–15.0) at 2 years postoperatively, showing stabilization from 6 weeks postoperatively onwards. In comparison, RSA subsidence of the non-radiographic subsiding stems (n = 25) was 0.5 mm (0.2 mm proximal translation to 3.6 mm subsidence) at 2 years postoperatively. 2 stems showed radiographic pedestal signs (both Reduced Distal). 1 stem dislocated during acquisition of radiographs directly postoperatively, which was non-surgically repositioned without recurring problems. Despite no incorrect sizing issues, 2 stems (Reduced Distal) did not have complete filling with respect to the femoral bone on the direct postoperative radiograph. Both stems subsided 6 weeks postoperatively (radiographic and RSA), but no underfilling was observed on the plain radiographs at this and later follow-up moments.

### Post-hoc migration analysis

LMM results without the 2 Reduced Distal stems with underfilled femoral canal showed almost identical subsidence results at 6 weeks postoperatively. Mean Y-axis translation for the Microplasty was –1.51 mm (CI –2.11 to –0.90) and for the Reduced Distal –1.46 mm (–2.01 to –0.90), with a mean design difference of –0.05 mm (CI –0.94 to 0.83) (Supplementary Table S3).

## Discussion

We showed initial subsidence and retroversion for both Microplasty and Reduced Distal stem designs, although this was likely not clinically relevant, and both designs maintained a stable position from 6 weeks to 2 years postoperatively. Post-hoc analysis showed that when accounting for the 2 underfilled patients the mean subsidence is almost identical, whereas the results for retroversion remained similar.

Our results are in accordance with other uncemented hip stem designs [7,9,10,21,22] and are currently considered a normal migration pattern. However, the 2-year postoperative subsidence for both designs was larger compared with the pooled subsidence for uncemented stems in a meta-analysis [9], larger than the pooled subsidence reported for uncemented stems in anterior-approach THA [10], and larger than the reported subsidence in recent RSA studies [7,21-23], all at 2 years postoperatively. De Waard et al. also reported individual Optimys short stems (3 out of 40) with > 5 mm subsidence measured with RSA [23]. This large subsidence was observed at 6 weeks postoperatively and remained the same throughout follow-up. The authors reasoned that the large initial migration in combination with an absence of continuous migration in these patients reflects suboptimal initial press fit during implantation, while good secondary fixation was achieved at later follow-up [23]. This study and our study indicate that short stems with metaphyseal fit may be quite forgiving.

A recent meta-analysis reported an approximate retroversion of 1.5° for uncemented anterior-approach THA (3 cohorts, unknown from meta-analysis which stem designs) [10]. This is similar to the retroversion of the Reduced Distal stems, but smaller than the retroversion of the Microplasty stems in the current study.

### Strengths

Strengths of the study were the randomized design and the use of RSA measurements to compare migration of 2 different stem designs. In addition, the use of LMM ensured the use of migration results from all follow-up moments despite missing data points.

### Limitations

First, we changed the primary outcome variable from MTPM to subsidence as subsidence is the most clinically relevant migration variable in hip migration studies. The change was made independent of data interpretation and the design of the study would have been identical if subsidence was the main outcome variable from study conception onwards. Although Evans [24] strongly recommends making protocol and trial amendments when a decision is made to change the primary outcome, this has not been done as the study was already concluded when this limitation became apparent.

Second, the absence of a proper sample size calculation for any of the outcome variables is a major shortcoming in the study design, although sufficient patients were included as recommended by Valstar et al. [14]. In combination with a larger than expected loss of available RSA results, our study is likely underpowered to detect a design difference in subsidence and retroversion. However, based on the migration rate for subsidence it seems justified to conclude that both designs do not further subside after initial settling.

Third, in our study both hip stems were randomized intentionally without considering femoral shape, though both stems are particularly designed for use in champagne-fluted femoral canals. 3 champagne-fluted femurs were included in this study, but without RSA results. This study included 3 stovepipe femurs as well, of which 1 (underfilled directly postoperatively), subsided the most. The other 2 stovepipe stems had no remarkable migration pattern. The post-hoc migration analysis showed that migration was similar for both designs, indicating that underfilling affects initial migration more than the femoral shape, which is supported by a previous study stratifying subsidence by femoral shape [7]. In the current study 35 of 46 patients with a study stem design were female, with equal ratios for both designs. Neither the post-menopausal status of the female patients nor the bone mineral density was registered, but previous studies reported a possible effect of bone mineral density on uncemented stem subsidence, retroversion, recovery time, and gait parameters [25,26]. The number of female patients could partly explain the large subsidence and retroversion observed in the current study.

Fourth, baseline RSA acquisitions were obtained after start of the rapid recovery program, and initial migration due to weightbearing as recommended cannot be assessed [8]. However, for uncemented hip stems, secondary stabilization is considered the best predictor for long-term aseptic loosening [9,10].

Fifth, the number of cases available for migration analysis was less than anticipated mainly for RSA technical reasons, which occurred more in the Microplasty group, though this seems unlikely to be related to the design and surgery of this particular stem as the Microplasty has a smaller volume compared with the Reduced Distal stem.

Sixth, all surgeries were performed by a single surgeon. Ideally results are included from different surgeons and different hospitals to improve generalizability of the results.

### Conclusion

We showed that the 2-year migration pattern of the shortened stem and reduced distal stem used in this MIS-ASI primary hip replacement study were similar. Both designs subsided and rotated into retroversion to a larger extent compared with other studies but the difference is likely not clinically relevant. Migration of both designs stabilized after 6 weeks. However, due to the small number of patients in this study and the large individual migration differences, one should be careful drawing general conclusions for the long-term success of these 2 stem designs in MIS-ASI primary hip replacement.

### Supplementary data

Supplementary Tables S1–S4 are available as Supplementary data on the article homepage, doi: 10.2340/17453674.2026.46551.

## Supplementary Material



## Notes

LAK wrote the original draft and performed project administration, data curation, investigation, formal analysis, and visualization; BLK reviewed and edited the manuscript and performed formal analysis, software, and validation; EG reviewed and edited the manuscript, performed data curation, and organized the outpatient clinic; CAMPB reviewed and edited the manuscript and performed conceptualization, surgery, and data curation; RGHHN reviewed and edited the manuscript.

The authors would like to thank all patients who dedicated their time to this study, and the staff from the department of radiology who assisted in acquiring all the RSA images.

Handling co-editors: Keijo Mäkelä and Jonas Ranstam

*Acta* thanks Hannu T Aro and Olof Sandberg for help with peer review of this manuscript.
