# Supplementary material for: Similar 2 year migration patterns for a shortened and a distally reduced stem design in uncemented total hip arthroplasty: a randomized controlled trial using radiostereometric analysis
Source: Acta Orthop. 2026 Aug 31;97:568–75. doi: 10.2340/17453674.2026.46551 (PMC13527608; doi:10.2340/17453674.2026.46551)
Supplement: Supplementary file 1 [file ActaO-97-46551-s1.pdf]

# Supplementary Data

Table S1a. Mean (SD) of unmodelled hip stem migration results of the Microplasty stem

| | | **Translation** (mm) | | | **Rotation** (°) | | | **MTPM (mm)** |
|---|---|---|---|---|---|---|---|---|
| **Follow-up** | | **X** | **Y** | **Z** | **X** | **Y** | **Z** | |
| 6 weeks (n = 15) | Mean | 0.28 | –1.51 | –0.47 | –0.07 | 2.85 | –0.34 | 3.57 |
| | SD | 0.41 | 1.43 | 0.54 | 0.62 | 2.23 | 0.97 | 2.35 |
| 1 year (n = 15) | Mean | 0.32 | –1.49 | –0.30 | –0.00 | 2.94 | –0.27 | 3.45 |
| | SD | 0.43 | 1.42 | 0.40 | 0.83 | 1.89 | 1.01 | 2.23 |
| 2 years (n = 14) | Mean | 0.24 | –1.50 | –0.15 | 0.04 | 2.54 | –0.29 | 3.28 |
| | SD | 0.45 | 1.46 | 0.44 | 0.80 | 2.44 | 1.01 | 2.38 |

Translation in millimeter (mm) and rotation in degrees (°) along and about the X-, Y- and Z-axes;

MTPM = maximum total point motion

Table S1b. Mean (SD) of unmodelled hip stem migration results of the Reduced Distal stem

| | | **Translation** (mm) | | | **Rotation** (°) | | | **MTPM (mm)** |
|---|---|---|---|---|---|---|---|---|
| **Follow-up** | | **X** | **Y** | **Z** | **X** | **Y** | **Z** | |
| 6 weeks (n = 19) | Mean | –0.24 | –2.41 | –0.22 | –0.51 | 1.47 | 0.15 | 3.78 |
| | SD | 1.67 | 3.56 | 0.43 | 0.80 | 2.46 | 1.09 | 4.16 |
| 1 year (n = 20) | Mean | –0.20 | –2.38 | –0.29 | –0.60 | 1.73 | 0.12 | 4.03 |
| | SD | 1.60 | 3.57 | 0.52 | 0.86 | 2.03 | 1.17 | 4.13 |
| 2 years (n = 19) | Mean | –0.26 | –2.31 | –0.26 | –0.54 | 1.43 | 0.20 | 3.90 |
| | SD | 1.69 | 3.66 | 0.55 | 0.93 | 2.20 | 1.09 | 4.17 |

Translation in millimeter (mm) and rotation in degrees (°) along and about the X-, Y- and Z-axes;

MTPM = maximum total point motion

Table S2. Median (IQR) of HHS and PROM scores at each follow-up for the Microplasty and Reduced Distal stems as implanted

| | | Follow-up | | | |
|---|---|---|---|---|---|
| **PROM** | **Design<br>(n = per follow-up)** | **Preoperative** | **6 week** | **1 year** | **2 years** |
| **HHS** | Microplasty<br>(n = 25; 25; 25; 24) | 60.0<br>(56.0–69.0) | 82.0<br>(69.5–88.5) | 99.0<br>(93.0–100.0) | 100.0<br>(97.0–100.0) |
| | Reduced Distal<br>(n = 21; 20; 21; 21) | 60.0<br>(54.5–75.5) | 81.5<br>(75.3–88.5) | 98.0<br>(91.5–100.0) | 100.0<br>(97.00–100.0) |
| **OHS** | Microplasty<br>(n = 25; 24; 25; 24) | 25.0<br>(21.5–31.5) | 39.5<br>(33.3–43.0) | 46.0<br>(43.0–48.0) | 48.0<br>(44.3–48.0) |
| | Reduced Distal<br>(n = 20; 21; 20; 20) | 26.5<br>(20.8–30.0) | 38.0<br>(36.0–40.0) | 43.5<br>(40.25–47.0) | 46.5<br>(42.3–48.0) |
| **EQ5D** | Microplasty<br>(n = 25; 25; 25; 24) | 0.69<br>(0.30–0.78) | 0.81<br>(0.78–0.90) | 1.00<br>(0.84–1.00) | 1.00<br>(0.88–1.00) |
| | Reduced Distal<br>(n = 20; 20; 18; 20) | 0.69<br>(0.48–0.78) | 0.84<br>(0.78–0.97) | 1.00<br>(0.81–1.00) | 1.00<br>(0.85–1.00) |
| **EQ5D-Health** | Microplasty<br>(n = 25; 24; 25; 24) | 60.0<br>(50.0–70.50) | 80.0<br>(70.0–90.0) | 80.0<br>(71.3–90.0) | 80.0<br>(75.0–90.0) |
| | Reduced Distal<br>(n = 20; 21; 20; 20) | 70.0<br>(51.3–77.3) | 80.0<br>(70.0–90.0) | 80.0<br>(75.3–91.8) | 86.5<br>(71.3–90.0) |
| **FJS** | Microplasty<br>(n = n/a; 25; 25; 23) | n.a. | 48.0<br>(19.0–75.0) | 75.0<br>(56.0–92.0) | 92.0<br>(69.0–98.0) |
| | Reduced Distal<br>(n = n/a; 21; 20; 19) | n.a. | 35.0<br>(26.0–66.0) | 61.5<br>(38.75–91.0) | 88.0<br>(58.0–98.0) |
| **HOOS-Pain** | Microplasty<br>(n = 25; 25; 25; 24) | 45.0<br>(35.0–52.5) | 82.5<br>(66.3–92.5) | 97.5<br>(91.3–100.0) | 100.0<br>(92.5–100.0) |
| | Reduced Distal<br>(n = 21; 21; 21; 20) | 42.50<br>(35.0–50.0) | 85.0<br>(69.4–90.0) | 95.0<br>(90.0–100.0) | 95.0<br>(85.6–100.0) |
| **HOOS-Symptoms** | Microplasty<br>(n = 25; 25; 25; 24) | 40.0<br>(32.5–52.5) | 75.0<br>(60.0–87.5) | 90.0<br>(80.0–97.5) | 95.0<br>(81.3–100.0) |
| | Reduced Distal<br>(n = 21; 21; 21; 20) | 40.0<br>(30.0–52.5) | 70.0<br>(62.5–85.0) | 90.0<br>(72.5–95.0) | 87.5<br>(81.3–95.0) |
| **HOOS-ADL** | Microplasty<br>(n = 25; 25; 25; 24) | 44.1<br>(33.1–59.6) | 85.3<br>(71.1–94.7) | 92.7<br>(85.2–100.0) | 98.5<br>(87.1–100.0) |
| | Reduced Distal<br>(n = 21; 21; 21; 20) | 42.7<br>(33.1–47.0) | 81.3<br>(75.7–87.5) | 92.7<br>(80.9– 97.1) | 92.7<br>(85.7–98.5) |
| **HOOS-Sports** | Microplasty<br>(n = 25; 24; 23; 22) | 18.8<br>(6.3–31.3) | 64.6<br>(50.0–75.0) | 81.3<br>(62.5–100.0) | 89.6<br>(67.2–100.0) |
| | Reduced Distal<br>(n = 21; 21; 21; 20) | 18.8<br>(12.50–31.3) | 59.34<br>(41.2–75.0) | 71.9<br>(56.3–85.9) | 87.5<br>(67.8–93.78) |
| **HOOS-QoL** | Microplasty<br>(n = 25; 25; 25; 24) | 25.0<br>(15.6–37.5) | 62.5<br>(53.1–78.1) | 93.8<br>(75.0–100.0) | 93.8<br>(87.5–100.0) |
| | Reduced Distal<br>(n = 21; 21; 21; 20) | 25.0<br>(18.8–37.5) | 62.5<br>(50.0–71.9) | 81.3<br>(65.6–100.0) | 90.6<br>(75.0–98.4) |

IQR = interquartile range; HHS = Harris hip score; PROM = patient reported outcome measure; OHS = Oxford hip score; EQ5D = Euroqol 5 dimensions; FJS = forgotten joint score; HOOS = hip osteoarthritis outcome score; QOL = quality of live; n.a. = not applicable

Table S3. Modelled (LMM) mean migration results with 95% confidence interval (CI) and estimated mean difference (CI) of the patients with a good femoral canal fill (15 Microplasty and 18 Reduced Distal stems) (as implanted) at 6 weeks, 1 year, and 2 years follow-up, with respect to direct-postoperative follow-up

| | **Mean migration (CI)** | | |
|---|---|---|---|
| **Follow-up** | **Microplasty** | **Reduced Distal** | **Mean difference (CI) (Microplasty–Reduced Distal)** |
| **Tx** (mm) | | | |
| 6 weeks | 0.28 (0.14 to 0.43) | 0.12 (–0.01 to 0.25) | 0.16 (–0.05 to 0.37) |
| 1 year | 0.32 (0.17 to 0.46) | 0.11 (–0.02 to 0.24) | 0.21 (0.00 to 0.42) |
| 2 years | 0.24 (0.09 to 0.38) | 0.09 (–0.04 to 0.23) | 0.14 (–0.07 to 0.36) |
| **Ty** (mm) | | | |
| 6 weeks | –1.51 (–2.11 to –0.90) | –1.46 (–2.01 to –0.90) | –0.05 (–0.94 to 0.83) |
| 1 year | –1.49 (–2.09 to –0.88) | –1.43 (–1.98 to –0.88) | –0.05 (–0.94 to 0.83) |
| 2 years | –1.47 (–2.01 to –0.85) | –1.39 (–1.95 to –0.83) | –0.07 (–0.97 to 0.82) |
| **Tz** (mm) | | | |
| 6 weeks | –0.47 (–0.66 to –0.29) | –0.16 (–0.33 to 0.01) | –0.31 (–0.58 to –0.04) |
| 1 year | –0.30 (–0.48 to –0.11) | –0.24 (–0.41 to –0.07) | –0.05 (–0.32 to 0.22) |
| 2 years | –0.15 (–0.34 to 0.04) | –0.22 (–0.39 to –0.05) | 0.07 (–0.20 to 0.35) |
| **Rx** (°) | | | |
| 6 weeks | –0.07 (–0.34 to 0.21) | –0.28 (–0.53 to –0.03) | 0.21 (0.19 to 0.61) |
| 1 year | 0.00 (–0.27 to 0.27) | –0.38 (–0.63 to –0.13) | 0.38 (–0.01 to 0.77) |
| 2 years | –0.05 (–0.22 to 0.33) | –0.34 (–0.59 to –0.08) | 0.39 (–0.01 to 0.79) |
| **Ry** (°) | | | |
| 6 weeks | 2.85 (1.98 to 3.72) | 1.78 (0.98 to 2.59) | 1.07 (–0.21 to 2.34) |
| 1 year | 2.94 (2.07 to 3.81) | 1.96 (1.17 to 2.76) | 0.98 (–0.29 to 2.25) |
| 2 years | 2.57 (1.68 to 3.46) | 1.86 (1.05 to 2.67) | 0.71 (–0.58 to 2.00) |
| **Rz** (°) | | | |
| 6 weeks | –0.34 (–0.64 to –0.04) | –0.14 (–0.42 to 0.14) | –0.20 (–0.64 to 0.24) |
| 1 year | –0.27 (–0.57 to 0.04) | –0.19 (–0.47 to 0.09) | –0.08 (–0.52 to 0.36) |
| 2 years | –0.31 (–0.61 to 0.00) | –0.17 (–0.46 to 0.11) | –0.13 (–0.58 to 0.32) |

LMM = linear mixed model; Tx, Ty, Tz = translation in millimeter (mm) along the X-, Y- and Z-axes; Rx, Ry, Rz = rotation in degrees (°) about the X-, Y- and Z-axes

Table S4. Number, condition number and mean error of the femoral bone markers used in the RSA analysis. Note that information of the markers used in a marker-configuration model (MC-model) is included in the condition number information, but not in the mean error information

| | **Number of markers**<br>**median (range)** | **Condition number**<br>**mean (SD)** | **Mean error (mm)**<br>**mean (SD)** |
|---|---|---|---|
| Microplasty (n = 15) | 6 (5–9) | 90.9 (31.9) | 0.16 (0.07) [a] |
| Reduced distal (n = 20) | 6 (3–9) | 73.6 (29.7) | 0.15 (0.08) [b] |

Microplasty = Microplasty stem; Reduced distal = Reduced distal stem; SD = standard deviation

[a] based on 14 cases; [b] based on 16 cases

## Qualitative measures for RSA migration analysis

- Days (median (range)) between surgery and postoperative RSA radiograph acquisition, of all implanted study stems:
    - 1 day (1 to 14)
- Days (median (inter quartal range)) deviating from the exact 24-months postoperative day, of all patients with 24-months postoperative RSA and who are included in the final analysis:
    - 29 days later (2 days early to 46 days later)
